# UrQt: an efficient software for the Unsupervised Quality trimming of NGS data

**DOI:** 10.1186/s12859-015-0546-8

**Published:** 2015-04-29

**Authors:** Laurent Modolo, Emmanuelle Lerat

**Affiliations:** Université de Lyon; Université Lyon 1; CNRS; UMR 5558, Laboratoire de Biométrie et Biologie Evolutive, 43 bd du 11 novembre 1918, Villeurbanne cedex, 69622 France

**Keywords:** Quality control, Trimming, Next-generation sequencing, Unsupervised segmentation, Parallel computing

## Abstract

**Background:**

Quality control is a necessary step of any Next Generation Sequencing analysis. Although customary, this step still requires manual interventions to empirically choose tuning parameters according to various quality statistics. Moreover, current quality control procedures that provide a “good quality” data set, are not optimal and discard many informative nucleotides. To address these drawbacks, we present a new quality control method, implemented in UrQt software, for Unsupervised Quality trimming of Next Generation Sequencing reads.

**Results:**

Our trimming procedure relies on a well-defined probabilistic framework to detect the best segmentation between two segments of unreliable nucleotides, framing a segment of informative nucleotides. Our software only requires one user-friendly parameter to define the minimal quality threshold (phred score) to consider a nucleotide to be informative, which is independent of both the experiment and the quality of the data. This procedure is implemented in C++ in an efficient and parallelized software with a low memory footprint. We tested the performances of UrQt compared to the best-known trimming programs, on seven RNA and DNA sequencing experiments and demonstrated its optimality in the resulting tradeoff between the number of trimmed nucleotides and the quality objective.

**Conclusions:**

By finding the best segmentation to delimit a segment of good quality nucleotides, UrQt greatly increases the number of reads and of nucleotides that can be retained for a given quality objective. UrQt source files, binary executables for different operating systems and documentation are freely available (under the GPLv3) at the following address: https://lbbe.univ-lyon1.fr/-UrQt-.html.

**Electronic supplementary material:**

The online version of this article (doi:10.1186/s12859-015-0546-8) contains supplementary material, which is available to authorized users.

## Background

Next Generation Sequencing (NGS) technologies produce calling error probabilities for each sequenced nucleotide [1]. These probabilities, encoded as phred scores [2], are often high at the heads and tails of the reads, indicating low-quality nucleotides [3]. The presence of these unreliable nucleotides can result in missing or wrong alignments that can either increase the number of false negatives and false positives in subsequent analyses or can produce false *k*-mers in *de novo* assembly, increasing both the complexity of an assembly and the chance of producing misassemblies [4]. To remove these unreliable nucleotides and only work with informative nucleotides, most NGS data analyses start with a quality control (QC) step before any downstream analysis.

There are three types of approaches to address low-quality nucleotides. Classical QC strategies begin by removing an arbitrary number of nucleotides at the head and tail of each read, with tools such as the fastx_trimmer from the FASTX-Toolkit [5], after visualization of the per nucleotide sequence quality with tools such as FastQC [6]. Then, only reads of high quality are retained by other filters; for example, all reads with a given percentage of their length below a given phred score are excluded, using tools such as the fastq_quality_filter from FASTX-Toolkit. More recent approaches modify incorrectly called nucleotides by superimposing reads to each other and removing low frequency polymorphisms. This kind of approach often works using motifs of *k* nucleotides or *k*-mer to modify low frequency motifs based on the most frequent ones. However, this type of approach requires potentially high sequencing coverage (15x in the case of Quake [7] and 100x in the case of ALLPATHS-LG [8]) and cannot be applied to non-uniform sequencing experiments, such as RNA sequencing (RNA-Seq). Other approaches trim unreliable nucleotides at the head and tail of each read. With these approaches, one wants to find the best trade-off between removing unreliable nucleotides and keeping the longest reliable or informative subsequence for the entire read. Current trimming approaches rely on two types of algorithms: the running sum algorithm and the window-based algorithm (for a review see [4]). However, these algorithms only return good local cutting points for each read when it is necessary to find a good global cutting point to get the best trade-off between removing unreliable nucleotides and losing too much information. Moreover, most of these QC strategies rely heavily on manually chosen parameters that are difficult to interpret and cannot be easily automatized.

In the present work, we have developed the program UrQt to trim unreliable nucleotides at the heads and tails of NGS reads based on their phred scores. We define an informative segment as a segment whose nucleotides are on average informative and an informative nucleotide as a nucleotide with a quality score above a specified quality threshold. Our approach takes advantage of the expected shape of the calling error probability along each read (abruptly decreasing for the first nucleotides and slowly increasing with the size of the reads) to find the best partition between two segments of unreliable nucleotides to be trimmed –the head and the tail of the reads– and a central informative segment. UrQt implements an unsupervised segmentation algorithm to find the best trimming cut-points in each read by maximum likelihood. We use a probabilistic model to handle more naturally the trimming problem than other procedures using window-based or running sum algorithms [4]. Moreover, UrQt requires no data-dependent parameters and takes advantage of modern multicore achitectures, which makes it particularly interesting to be routinely applied for NGS reads in fastq or fastq.gz format [9] and attractive for the development of future analytical pipelines.

## Implementation

In this section, we present the probabilistic model that we use to find the best position to trim a read to increase its quality without removing more nucleotides than necessary. We also present an extension of this model for homopolymer trimming.

**Figure 1:**
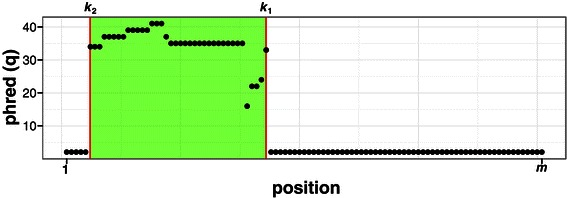
Quality trimming. Position of the cut-points *k*_1_ and *k*_2_ in a read. After trimming, the retained part corresponds to the section with a green background, which indicates an informative segment of nucleotides between *k*_1_ and *k*_2_.

A read is defined as a vector (*n*_1_,…,*n*_*m*_) of *m* nucleotides associated with a vector of phred scores (*q*_1_,…,*q*_*m*_). We want to find the best cut-point *k*_1_∈[1,*m*] in a read of length *m* between an informative segment for nucleotide *n*_*i*_,*i*∈[1,*k*_1_] and a segment of unreliable quality for nucleotide *n*_*i*_,*i*∈[*k*_1_+1,*m*] (Figure 1). Then, having found *k*_1_, we want to find the best cut-point *k*_2_∈[1,*k*_1_] between a segment of unreliable quality for nucleotide *n*_*i*_,*i*∈[1,*k*_2_−1] and an informative segment for nucleotide *n*_*i*_,*i*∈[*k*_2_,*k*_1_]. Given the shape of the calling error probability distribution, there is less signal to find *k*_1_ (the probability slowly increases at the extremity of the read) than *k*_2_ (abruptly decreases). Therefore, we want to have the highest number of nucleotides to support the choice of *k*_1_ when *k*_2_ can be found with a subsequence of the read (Figure 1).

**Figure 2:**
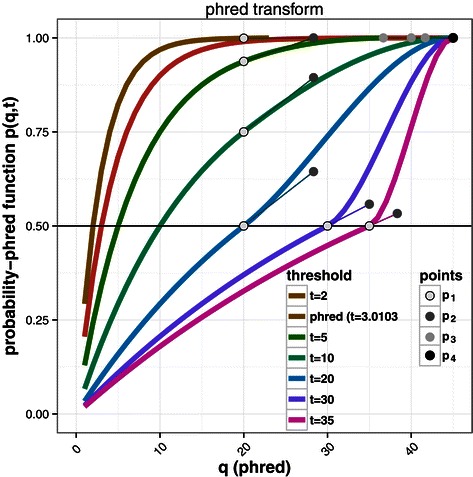
Probability-phred functions. *p*(*q*,*t*) according to the choice of *t*. The white, dark grey, light grey and black dots represent respectively the position of *p*_1_,*p*_2_,*p*_3_ and *p*_4_ for the corresponding probability-phred functions. Before *p*_1_ we have the $1-2^{\frac {-q}{t}}$ part of the function (B) and after *p*_1_ the *B*(*q* ^⋆^,*p*_1_,*p*_2_,*p*_3_,*p*_4_) part of the function (B).

With *q* the quality value of a nucleotide, the probability for this nucleotide to be correct is defined by:

(A) $$ p_{a}\left(q\right)=1-10^{\frac{-q}{10}}  $$

which gives, for example, a probability *p*_*a*_(*q*)=0.99 for a phred *q*=20 [2]. However, in QC, the word “informative” is typically defined as a phred score above a certain threshold and not the probability of calling the correct nucleotide. From a probabilistic point of view, we need to discriminate informative nucleotides (with *p*_*a*_(*q*)≥*p*_*a*_(*t*) and *t* a given threshold) from other nucleotides, rather than discriminate fairly accurate nucleotides (with *p*_*a*_(*q*)≥0.5) from the others. Therefore, we propose to define the probability of having an informative nucleotide as $p_{b}\left (q,t\right) = 1-2^{\frac {-q}{t}}$ with *t* the minimal phred score acceptable to be informative. This definition shifts the probability function such that for *q*=*t*, we have *p*_*b*_(*q*,*t*)=0.5. Therefore, at the threshold *t*, nucleotides with *p*_*b*_(*q*,*t*)≥0.5 are informative and the others are not. With *t*=3.0103, we go back to the classical phred function (Figure 2) in which *p*_*b*_(*q*,*t*)=*p*_*a*_(*q*).

With the function *p*_*b*_(*q*,*t*), low phred scores are associated with a low probability to be correct (*p*_*b*_(0,*t*)=0), but for *t*≤20 a high phred score does not correspond to a high probability to be correct (for example, *p*_*b*_(40,20)=0.75). Therefore, from a probabilistic point of view, unreliable nucleotides will have more weight than informative ones. To associate a high phred score with a high probability of having an informative nucleotide, we constrain this probability to reach 1 for a phred score of 45 by using the following spline function (Figure 2):

(B) $$ p\left(q,t\right) = \left\{ \begin{array}{ll} 1-2^{\frac{-q}{t}} & \text{if} \,\,q \leq \max(20,t),\\ B\left(q^{\star}, p_{1}, p_{2}, 1, 1\right) & \,\,\text{otherwise}\\ \end{array} \right.  $$

with *B*(*q* ^⋆^,*p*_1_,*p*_2_,*p*_3_,*p*_4_) the cubic Bezier curve starting at *p*_1_ toward *p*_2_ and arriving at *p*_4_ coming from the direction of *p*_3_ for *q* ^⋆^∈[0,1]. We have *p*_1_=1−2^− max(20,*t*)/*t*^, *p*_2_=*g*(1/3×(45− max(20,*t*))) with *g*(*q*) the tangent to the function $1-2^{\frac {-q}{t}}$ in max(20,*t*). We scale the Bezier curve to the interval [*t*,45] with *q* ^⋆^=(*q*−*t*)/(45−*t*). The constraint max(20,*t*) ensures that $\frac {d}{dq^{\star }} B\left (q^{\star }, p_{1}, p_{2}, p_{3}, p_{4}\right) < 0$ for *q* ^⋆^∈[0,1] (see Figure 2).

With the maximum likelihood framework, finding the position of the cut-point between a segment of informative nucleotides (*q*>*t*) and a segment of unreliable nucleotides (*q*<*t*) consists in estimating *k*_1_ by:

(C) $$ \widehat{k_{1}} = \arg\max\limits_{k} \prod\limits_{i=1}^{k} \frac{1}{k} f_{0}\left(n_{i}, t\right) \prod\limits_{i=k+1}^{m} \frac{1}{m-k-1} f_{1}\left(n_{i}, t\right)   $$

with *f*_0_(*n*_*i*_,*t*) the probability that the nucleotide *n*_*i*_ comes from the segment of informative nucleotides and *f*_1_(*n*_*i*_,*t*) the probability that the nucleotide *n*_*i*_ comes from the segment of unreliable nucleotides for a given *t*. Such that:

(D) $$ f_{0}\left(n_{i}, t\right) = p\left(q_{i}, t\right) \prod\limits_{N \in \Omega} \Pr(N)^{\mathbf{1}\left(n_{i} = N\right)}  $$

(E) $$ f_{1}\left(n_{i}, t\right) = \left(1-p\left(q_{i}, t\right)\right) \frac{1}{4}  $$

with **1**(*n*_*i*_=*N*) an indicator variable such that **1**(*n*_*i*_=*N*)=1 if the nucleotide *n*_*i*_ is equal to *N* and 0 otherwise, $\Pr (N)=\sum _{i=1}^{k} \mathbf {1}\left (n_{i}=N\right)/k \label {eq:pN}$ the probability to observe the nucleotide *N* between 1 and *k*, and *Ω* the standard IUB/IUPAC dictionary [10]. Pr(*N*)_*N*∈*Ω*_ and *k*_1_ are estimated with the complete data framework of the EM algorithm [11]. After finding $\widehat {k_{1}}$, we apply the same procedure on the interval $[1,\widehat {k_{1}}]$ to estimate the best cut-point *k*_2_ between a segment of unreliable nucleotides ahead of a segment of informative nucleotides. This double binary segmentation ensures to provide the best two cut-points for a given read [12].

For *p*(*q*,*t*)=*p*_*a*_(*q*), we can interpret the segment of informative nucleotides as a segment for which on average we are confident that a given nucleotide is the correct one, whereas the segment of unreliable nucleotides is composed of uninformative nucleotides in which on average any of the four nucleotides can be present at a given position. The cut-point *k*_1_ maximizes the probability that the nucleotides *n*_*i*_,*i*∈[1,*k*_1_] are informative and that nucleotides *n*_*i*_,*i*∈[*k*_1_,*m*] are not.

With our model, trimming nucleotides of unreliable quality is somewhat similar to removing homopolymers from the extremities of the reads. The task of removing homopolymers, such as poly*A* tails in RNA-Seq experiments, is not trivial, because the quality of a given nucleotide decreases both at the end of the read and with the size of the homopolymer. Therefore, because the number of incorrectly called nucleotides increases, we are less likely to observe *A*s at the end of the poly*A* tail. UrQt implements a procedure for the unsupervised trimming of poly*N* with a straightforward modification of equation (E) such that:

(F) $$ f_{1}\left(n_{i}, t\right) = p_{a}\left(q_{i}, t\right)^{\mathbf{1}\left(n_{i} = A\right)} \left(\left(1-p_{a}\left(q_{i}, t\right)\right)\frac{1}{4}\right)^{\mathbf{1}\left(n_{i} \ne A\right)}  $$

in which we can replace *A* by any letter of the standard IUB/IUPAC dictionary. With this definition of *f*_1_, we consider the calling error probability of the nucleotide at position *i* if *n*_*i*_=*A* or if *n*_*i*_≠*A*, the probability that the nucleotide could have been an *A*.

## Results and discussion

To assess the performance of our approach, we compared the performance of UrQt to other publicly available programs on different NGS data sets (see Table 1). The quality of the data generated during an NGS experiment can vary greatly depending on the type of data (DNA or RNA) and the sequencing pipeline. To analyze these two types of data on the same genome, we chose paired-end RNA and paired-end DNA sequencing experiments from the species *Drosophila melanogaster*. For this species, the DNA sample quality quickly drops at the end of the reads (see Additional file 1), and the RNA sample presents a large variability of quality among its reads. We also included in our analysis four other data sets from four different species which are the same ones as used in the comparative study of Del Fabbro et al. [4]. One single-end RNA sample from the species *Homo sapiens* of poor overall quality and one single-end RNA sample of good overall quality from the species *Arabidopsis thaliana*. For the DNA sample, we used one paired-end sample from the species *Prunus persica* of excellent overall quality and one paired-end DNA sample from the species *Saccharomyces cerevisiae* of average quality. Finally, we also included one paired-end RNA sample from the species *Homo sapiens* of overall good quality. The seven data sets (Table 1) were downloaded from the NCBI website. Rather than using the complete data set, we uniformly sampled 500,000 reads from each experiment using the software fastq_sampler.py (available at https://github.com/l-modolo/fastq_sampler), to speed-up the computation and work with comparable reads number for each sample.

**Table 1:** **NGS data sets used for testing**

**Accession**	**Species**	**Sample**	**Paired-end**	**Read size**	**Reference**
**number**		**type**		**(bp)**	**genome**
SRR002073	*Homo sapiens*	RNA	no	33	hg19
SRR521463	*Homo sapiens*	RNA	yes	75	hg19
SRR420813	*Arabidopsis thaliana*	RNA	no	83	TAIR10
SRX150254	*Prunus persica*	DNA	yes	100	1.22
SRR452441	*Saccharomyces cerevisiae*	DNA	yes	100	EF4
SRR988074	*Drosophila melanogaster*	DNA	yes	101	5.41
SRR919326	*Drosophila melanogaster*	RNA	yes	101	5.41

For testing purposes, we choose the better trimming programs, according to their performances in the study of Del Fabbro et al. [4] and representing both running sum algorithms (Cutadapt [13], which implement the algorithm proposed for BWA [14]) and sliding-windows algorithms (Trimmomatic [15] and Sickle [16]). The different programs were compared on two points: the overall quality of the resulting trimmed data set and the number of reads mapped on the corresponding reference genome with Bowtie2 [17] for different quality thresholds. For the analyses presented in this work, we used the latest available versions of Cutadapt (version 1.4.1), Trimmomatic (version 0.32) and Sickle [16] (version 1.290). The value of the quality threshold *t* for the three programs, corresponded respectively to the parameter *–t* for UrQt, *–q* for Cutadapt and Sickle and *SLIDINGWINDOW:4:t* for Trimmomatic. All the other parameters were set to default values, except for the minimum read length that was set to 1 bp. All quality figures were generated with FastQC [6] and the quality statistics were computed using R [18] and the FASTX-Toolkit [5].

### Consistency of the trimming procedures

**Figure 3:**
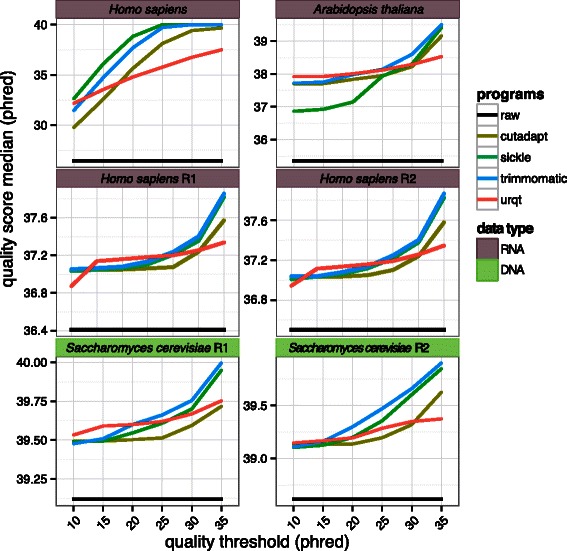
Quality of the trimmed data for each software. Performances of different trimming algorithms in terms of the median quality (phred) of the resulting trimmed data set for different quality thresholds. The choices of *t* correspond to the parameter *–t* for UrQt, *–q* for Cutadapt and Sickle and *SLIDINGWINDOW:4:t* for Trimmomatic. The black line corresponds to raw (untrimmed) data, and R1 and R2 correspond to the two ends of paired-end data.

It is expected that the quality in the trimmed data set will increase with the quality threshold up to a certain saturation point. We computed the median quality (phred) in the trimmed data for different quality thresholds (Figure 3, and Additional file 2 for the seven data sets). We observed from this comparison that except for UrQt, all other programs failed to produce a stable relationship between the chosen quality threshold and the resulting median quality score across different samples. For example, we observed a logarithmic-like relationship between the quality threshold and the median for data sets of overall poor quality, such as the *H. sapiens* data of overall poor quality, and an exponential-like relationship for data sets of overall good quality, such as the *A. thaliana* and the *S. cerevisiae* data (Figure 3). These different types of relationships indicate that an increase of the threshold does not have the same effect from one data set to another, and that this effect also depends on the value of the threshold. However, with UrQt, we observe a stable relationship between the threshold and the median quality that is representative of more consistent cutting-points. With a stable relationship between the threshold and the quality of the trimmed data set, it is thus possible to set the quality threshold beforehand according to a targeted quality and independently of the data.

### Optimality of the trimming procedures

**Figure 4:**
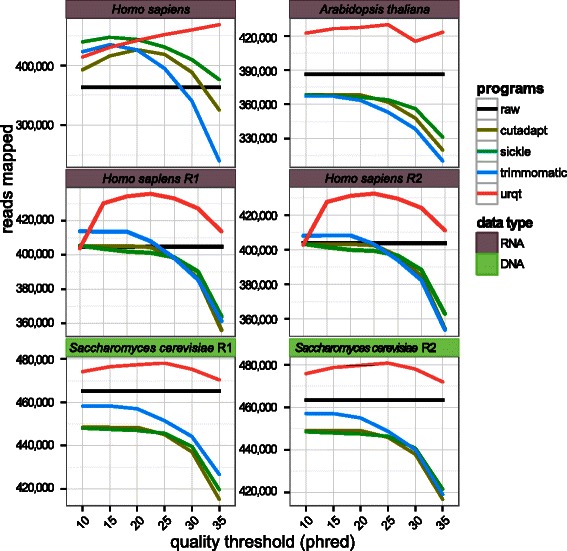
Remaining information in the trimmed data for each software. Mapping performances for different quality threshold. The choice of *t* corresponds to the parameter *–t* for UrQt, *–q* for Cutadapt and Sickle and *SLIDINGWINDOW:4:t* for Trimmomatic. The black line corresponds to raw (untrimmed) data, and R1 and R2 correspond to the two ends of paired-end data.

Although increasing the quality of a data set by trimming nucleotides of poor quality is easy, the remaining difficulty lies in minimizing the information (nucleotides) lost in the process. A simple metric to evaluate this trade-off is the number of trimmed reads that can be mapped on the corresponding reference genome. With better quality information after trimming, we expect an increase of the number of mapped reads, whereas by removing too many nucleotides, we expect less information and thus a decrease in the number of mapped reads. For the mapping procedure, we used Bowtie2 [17] (version 2.2.2) (with default parameters and the *–very-sensitive* option) and the genome indexes available from the igenome project (see Table 1 and Additional file 3 for the version). For the paired-end data, each end was mapped independently. We examined the number of mapped reads on the corresponding reference genomes (Table 1) for different quality thresholds (Figure 4 and Additional file 4 for the seven data sets). The same mapping procedure was also performed using BWA [14] (version 0.7.10) (with default parameters) (Additional file 3). We observed that UrQt was the only software that consistently increased the number of mapped reads for all data sets. The other programs provided the desired effect only for data sets of overall poor quality, such as for the single-end *H. sapiens* data (SRR002073), and produced worse results than those obtained by mapping the raw data for data sets of better quality (Figure 4). For the single-end *H. sapiens* data, we observed that UrQt better respected the chosen threshold, thus producing worst results than the other programs for the low quality threshold. For example, with this dataset and a threshold of 5, we expect a large number of reads with an average quality slightly above 5 which are difficult to map. This respect of the threshold can also be seen for the paired-end *H. sapiens* data (SRR521463) or the *D. melanogaster* RNA data (SRR919326) and a low threshold of 5 where UrQt is the only program that produces results comparable to the raw data (see Additional file 4).

For data sets of excellent quality, such as *P. persica* (see Additional file 4), all the trimming programs except for UrQt deteriorated the mapping performances compared with the ones obtained by mapping the raw data. This result provides additional evidence of better trimming cut-points identified by UrQt compared with the ones found by other procedures that remove too many nucleotides for data sets of excellent quality.

When considering the output of a mapping software, we can discriminate between reads, which map to a unique position and reads, which map to multiple positions. The number of reads mapping at multiple positions depends on three factors: the number of reads associated with repetition, the sensitivity of the mapping procedure (we can expect more reads mapping at multiple positions when allowing for more missmatches and gaps), and the information contained in the reads. Thus with trimming procedures, the information loss of over-trimming could lead to an increase of the number of reads mapping at multiple positions. This over-trimming effect can be seen with Cutadapt, Trimmomatic and Sickle for high threshold values (superior to 20) (see Additional file 3 for the results with Bowtie2 and BWA). However, with UrQt, the number of reads mapping to unique position increase with the choice of the threshold which is also consistent with better cut-point. These results hold for every dataset with the exception of the *H. sapiens* RNA sample of poor overall quality (SRR002073) for which removing a large number of uninformative nucleotides also correspond to removing a large number of reads.

Overall, the results obtained with UrQt correspond to the expected results for a trimming procedure and a given quality threshold in opposition to the other programs in our test panel (see Additional file 3 and 5). The output of UrQt depends on the choice of *t* that defines an informative sequence for which we expect nucleotides to have a phred score above this threshold. Contrary to current methods in which the choice of the threshold is set according to the quality of the data, the UrQt *–t* parameter only depends on the goal of the analysis (SNP calling, *de novo*-assembly, mapping, etc.).

## Conclusions

UrQt is a new tool for the key QC step of any NGS data analysis to trim low-quality nucleotides and poly*A* tails from reads in fastq or fastq.gz format with an efficient C++ implementation. By finding the best segmentation to delimit a segment of informative nucleotides, UrQt greatly increases the number of reads and of nucleotides that can be retained for a given quality objective. Using this software should provide a significant gain for many NGS applications. Moreover, the consistency of our trimming procedure with the quality of the trimmed data set for a given quality threshold, will allow for better automation of the trimming step in a pipeline. We also provide a galaxy wrapper for UrQt to facilitate its integration in existing pipelines developed on this platform [19-21]. Finally, with our simple probabilistic model for the trimming of NGS data, we hope that users will have a better grasp on the quality threshold *–t* to obtain the largest trimmed data set with the required quality.

## Availability and requirements

**Project name:** UrQt**Project home page:** https://lbbe.univ-lyon1.fr/-UrQt-.html **Operating system(s):** Platform independent**Programming language:** C++**Other requirements:** zlib and c++0x compiler**License:** GNU GPLv3**Any restrictions to use by non-academics:** GNU GPLv3

## Additional files

Additional file 1
**Quality analysis of the seven NGS samples.** Quality analysis of the seven NGS samples (Table 1) with the FastQC software.

Additional file 2
**Quality of the trimmed data for each programs.** Performances of different trimming algorithms in terms of the median quality (phred) of the resulting trimmed data set for different quality thresholds. The choice of *t* corresponds to the parameter *–t* for UrQt, *–q* for Cutadapt and Sickle and *SLIDINGWINDOW:4:t* for Trimmomatic. The black line corresponds to raw (untrimmed) data, and R1 and R2 correspond to the two ends of paired-end data. This figure complements the Figure 3 with the seven data sets (Table 1).

Additional file 3
**Mapping performances for the four tested programs.** Mapping performances for different quality threshold with the four tested programs and the seven NGS sample (Table 1). Mapping results with Bowtie2 [17] and BWA [14].

Additional file 4
**Remaining information in the trimmed data for each programs.** Mapping performances for different quality threshold. The choice of *t* correspond to the parameter *–t* for UrQt, *–q* for Cutadapt and Sickle, and *SLIDINGWINDOW:4:t* for Trimmomatic. The black line corresponds to raw (untrimmed) data, and R1 and R2 correspond to the two ends of paired-end data. This figure complements the Figure 4 with the seven data sets (Table 1).

Additional file 5
**Quality analysis of the seven NGS samples for the four tested programs.** Quality analysis of the output of the four programs for the seven NGS samples and different quality thresholds (Table 1) with the FastQC [6] software.
