# Supplementary material for: UrQt: an efficient software for the Unsupervised Quality trimming of NGS data
Source: BMC Bioinformatics. 2015 Apr 29;16:137. doi: 10.1186/s12859-015-0546-8 (PMC4450468; doi:10.1186/s12859-015-0546-8)
Supplement: Additional file 1 — Quality analysis of the seven NGS samples. Quality analysis of the seven NGS samples (Table 1) with the FastQC software. [file 12859_2015_546_MOESM1_ESM.pdf]

Quality analysis of the seven NGS samples

December 19, 2014

Quality analysis of the seven NGS samples (Table 1) with the FastQC software [1].

Table 1: **NGS data sets used for testing**

Accession number	Species	sample type	paired-end	read size (bp)	reference genome
SRR002073	*Homo sapiens*	RNA	no	33	hg19
SRR521463	*Homo sapiens*	RNA	yes	75	hg19
SRR420813	*Arabidopsis thaliana*	RNA	no	83	TAIR10
SRX150254	*Prunus persica*	DNA	yes	100	1.22
SRR452441	*Saccharomyces cerevisiae*	DNA	yes	100	EF4
SRR988074	*Drosophila melanogaster*	DNA	yes	101	5.41
SRR919326	*Drosophila melanogaster*	RNA	yes	101	5.41

Contents

1 *Homo sapiens* RNA sample (SRR002073) 3
1.1 Per base sequence quality . . . 3
1.2 Per sequence quality scores . . . 3
1.3 Per base sequence content . . . 3

2 *Homo sapiens* RNA sample (SRR521463) 4
2.1 Per base sequence quality . . . 4
2.2 Per sequence quality scores . . . 4
2.3 Per base sequence content . . . 4

3 *Arabidopsis thaliana* RNA sample (SRR420813) 5
3.1 Per base sequence quality . . . 5
3.2 Per sequence quality scores . . . 5
3.3 Per base sequence content . . . 5

4 *Prunus persica* DNA sample (SRX150254) 6
4.1 Per base sequence quality . . . 6
4.2 Per sequence quality scores . . . 6
4.3 Per base sequence content . . . 6

5 *Saccharomyces cerevisiae* DNA sample (SRR452441) 7
5.1 Per base sequence quality . . . 7
5.2 Per sequence quality scores . . . 7
5.3 Per base sequence content . . . 7

6 *Drosophila melanogaster* RNA sample (SRR919326) 8
6.1 Per base sequence quality . . . 8
6.2 Per sequence quality scores . . . 8
6.3 Per base sequence content . . . 8

7 *Drosophila melanogaster* DNA sample (SRR988074) 9
7.1 Per base sequence quality . . . 9
7.2 Per sequence quality scores . . . 9
7.3 Per base sequence content . . . 9

1 *Homo sapiens* RNA sample (SRR002073)

1.1 Per base sequence quality

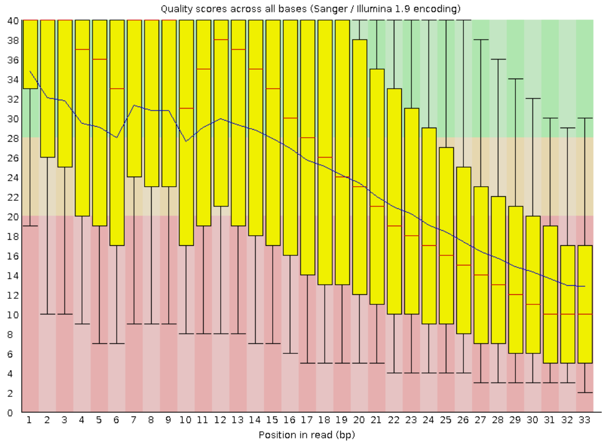

1.2 Per sequence quality scores

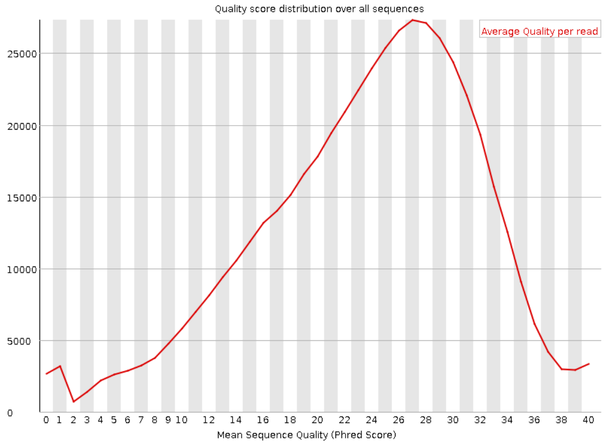

1.3 Per base sequence content

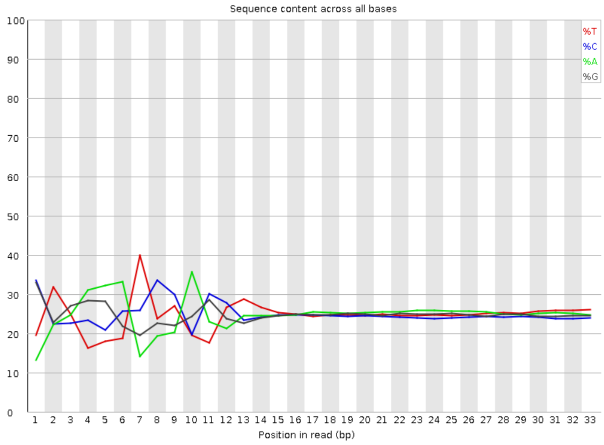

W

2 *Homo sapiens* RNA sample (SRR521463)

2.1 Per base sequence quality

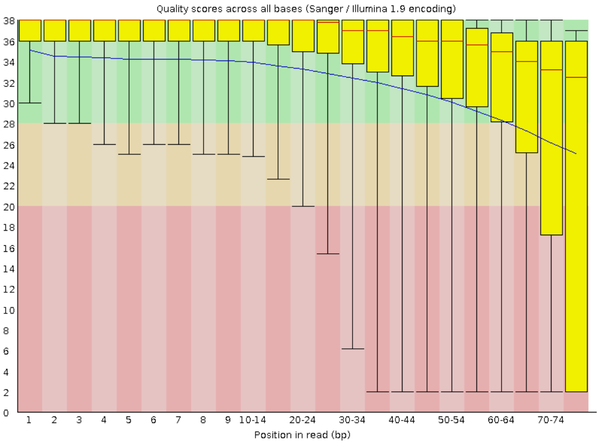

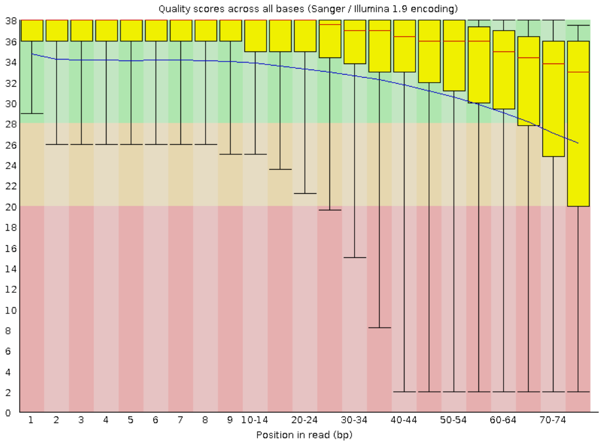

2.2 Per sequence quality scores

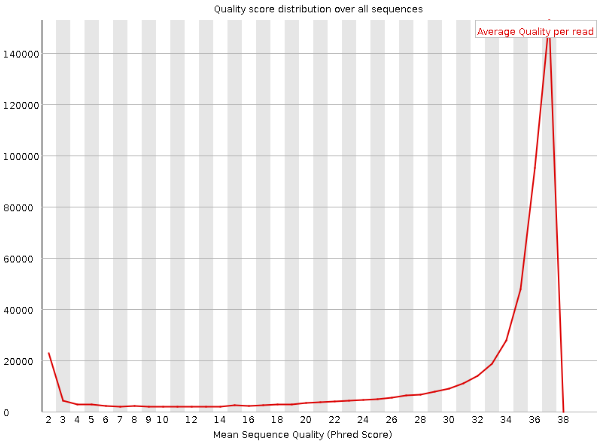

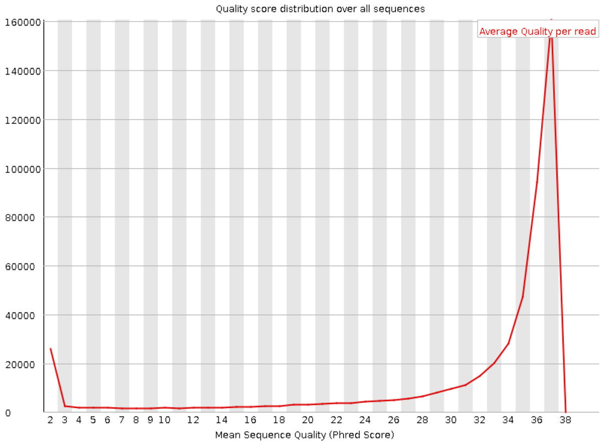

2.3 Per base sequence content

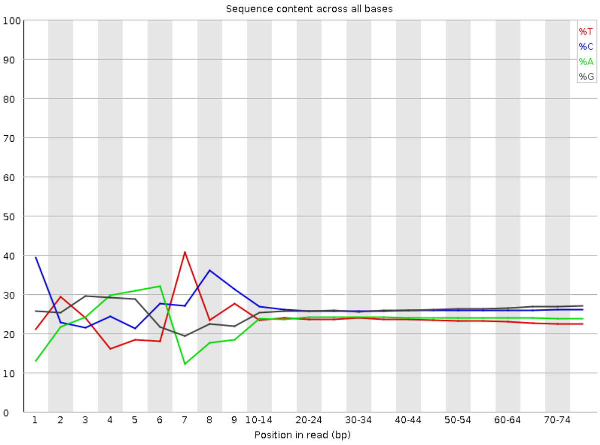

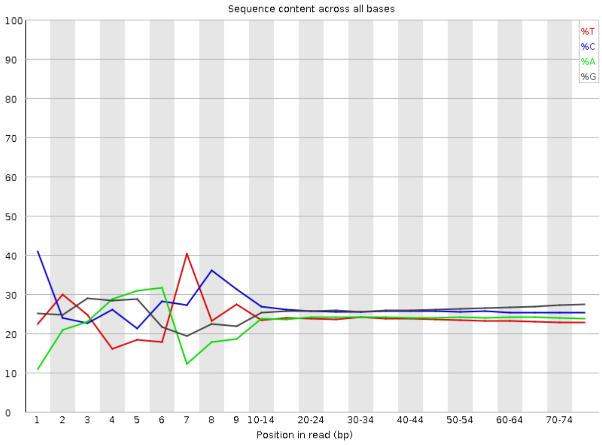

3 *Arabidopsis thaliana* RNA sample (SRR420813)

3.1 Per base sequence quality

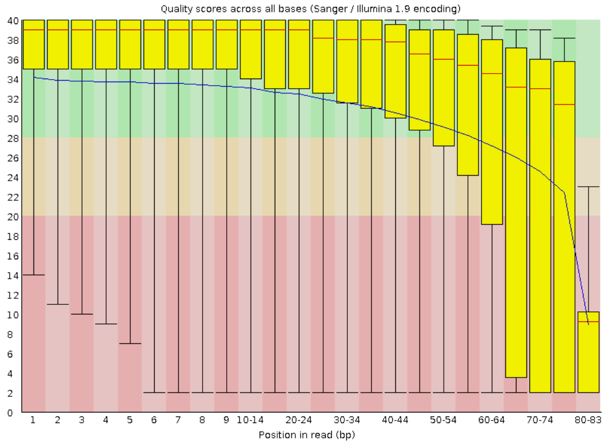

3.2 Per sequence quality scores

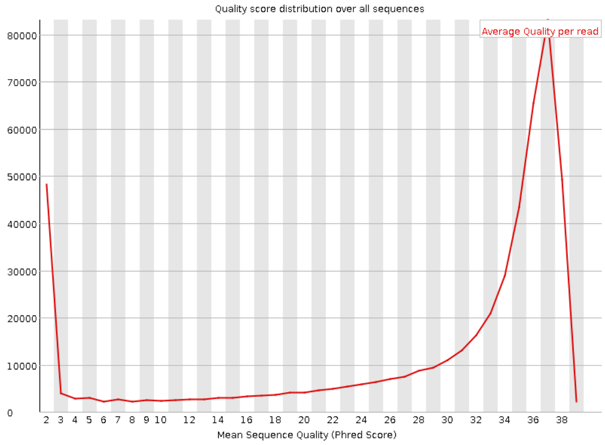

3.3 Per base sequence content

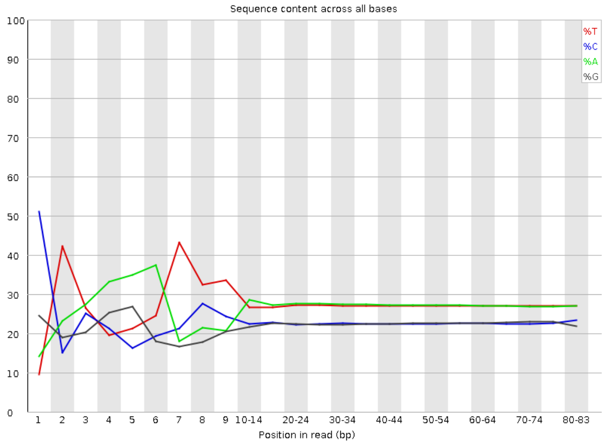

4 *Prunus persica* DNA sample (SRX150254)

4.1 Per base sequence quality

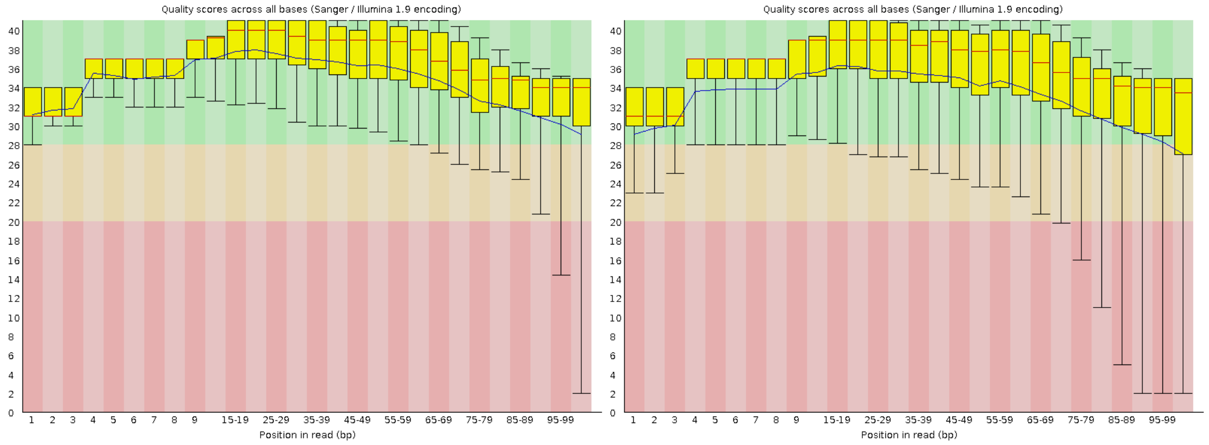

4.2 Per sequence quality scores

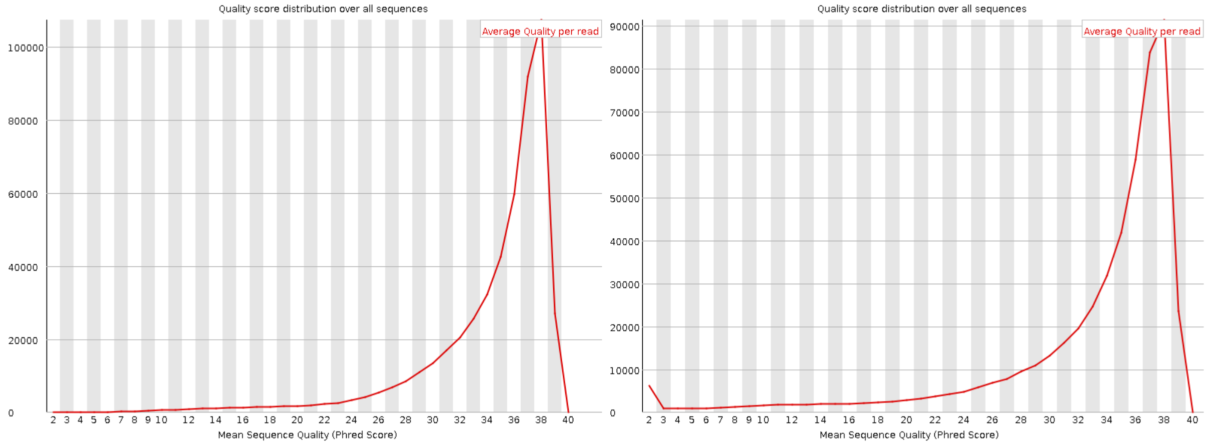

4.3 Per base sequence content

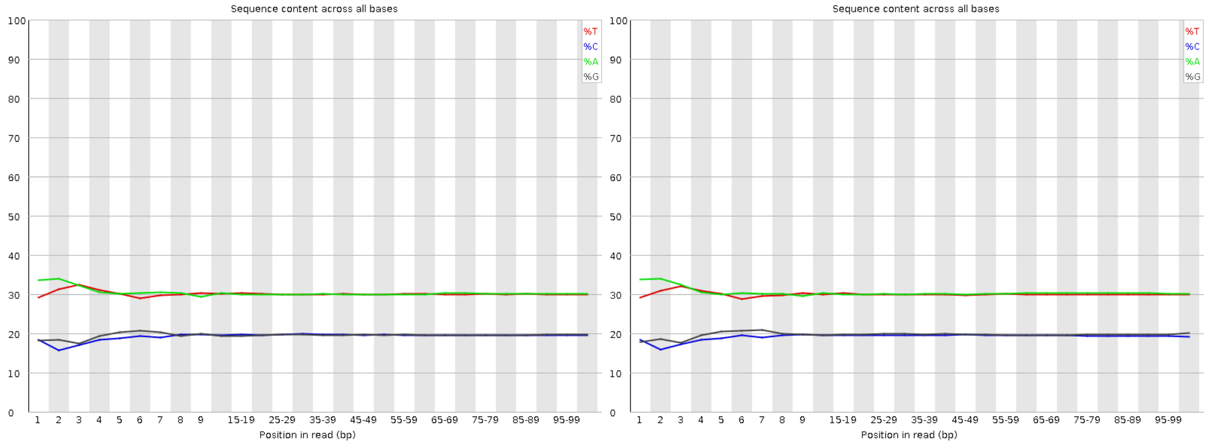

5 *Saccharomyces cerevisiae* DNA sample (SRR452441)

5.1 Per base sequence quality

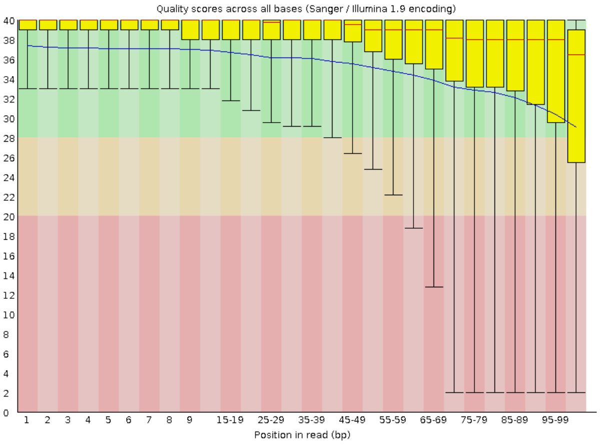

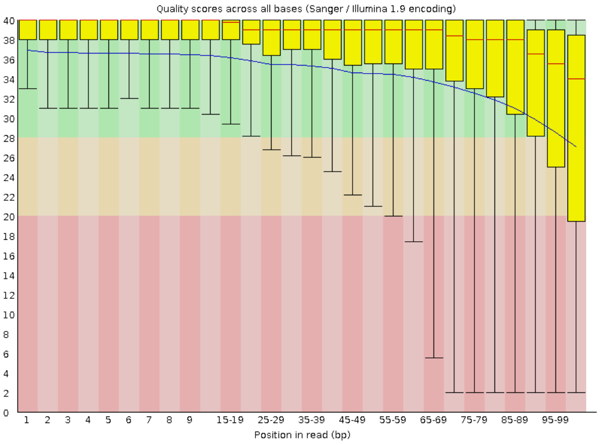

5.2 Per sequence quality scores

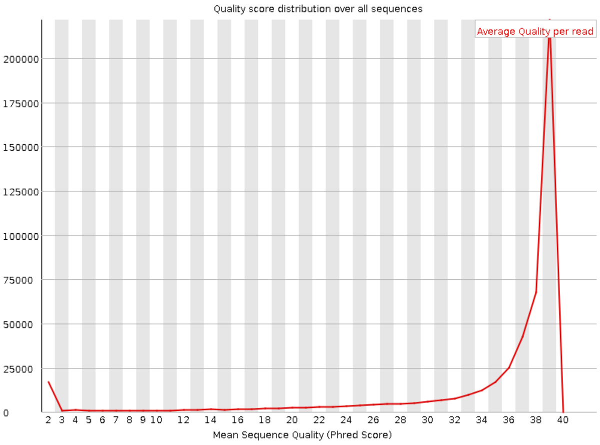

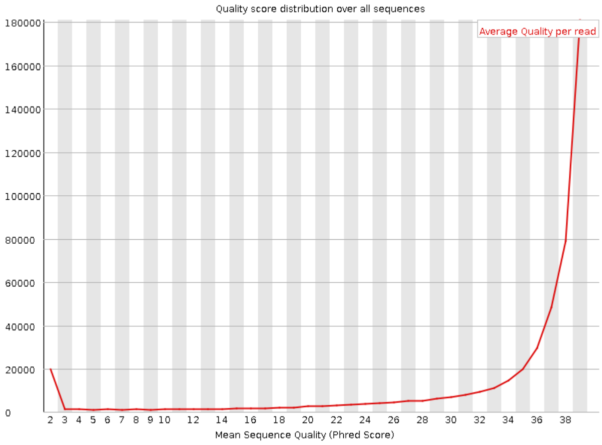

5.3 Per base sequence content

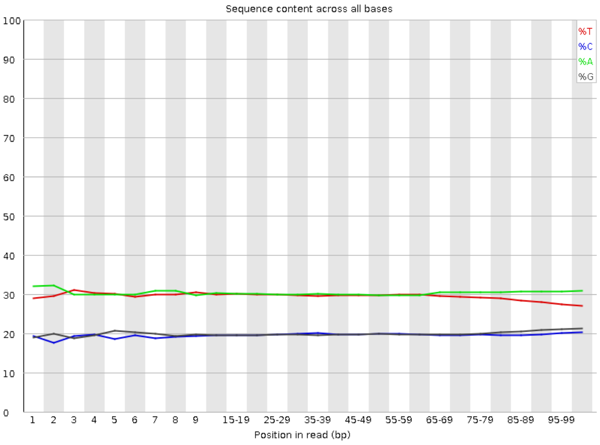

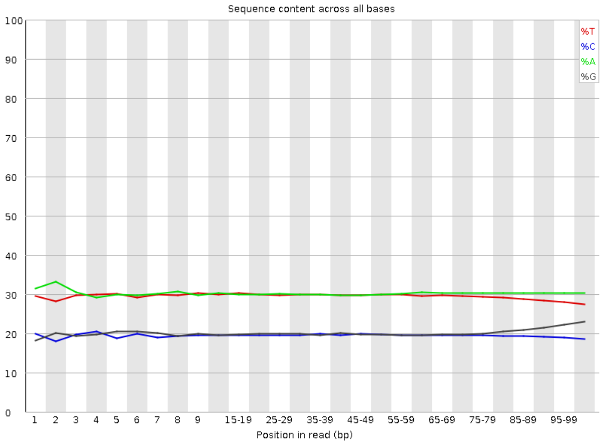

6 *Drosophila melanogaster* RNA sample (SRR919326)

6.1 Per base sequence quality

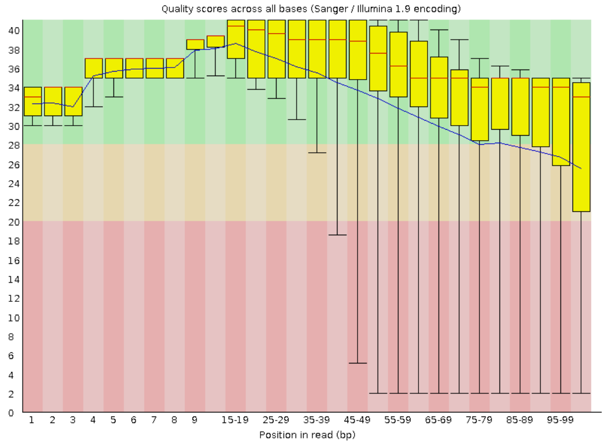

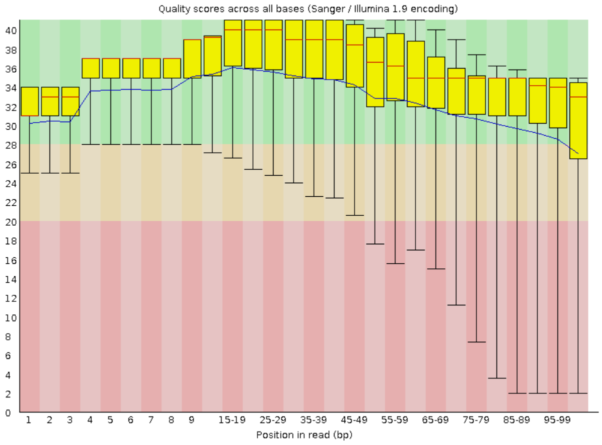

6.2 Per sequence quality scores

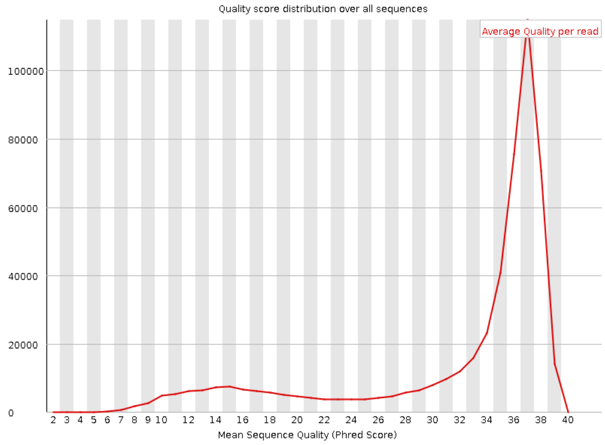

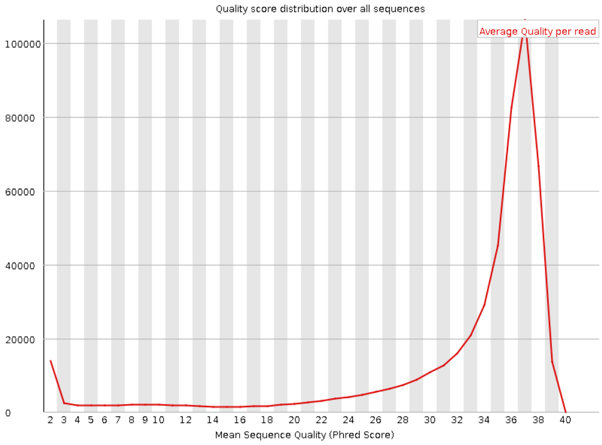

6.3 Per base sequence content

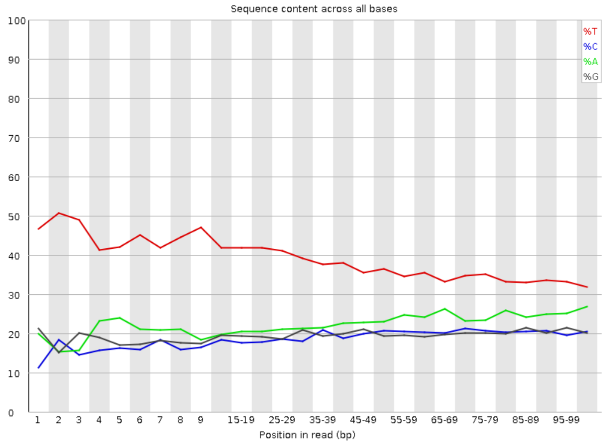

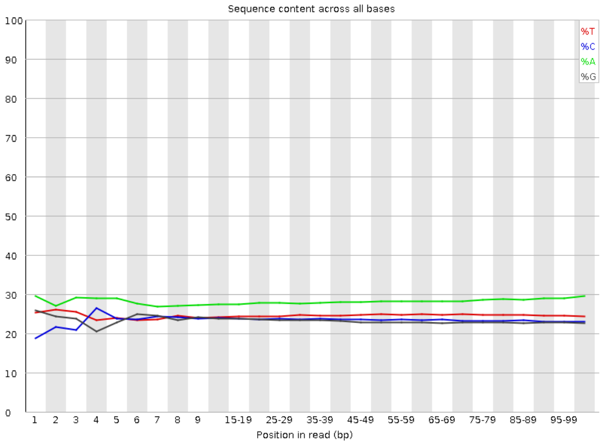

7 *Drosophila melanogaster* DNA sample (SRR988074)

7.1 Per base sequence quality

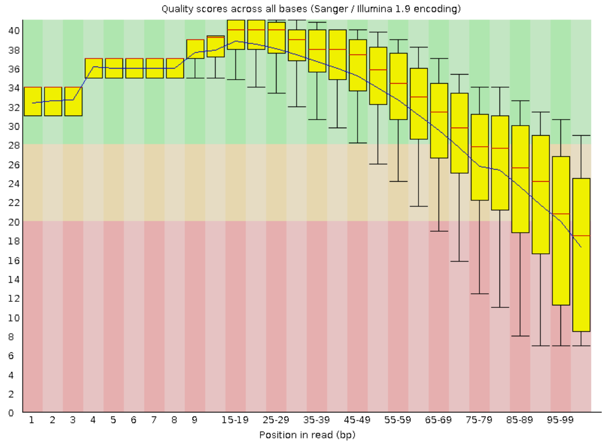

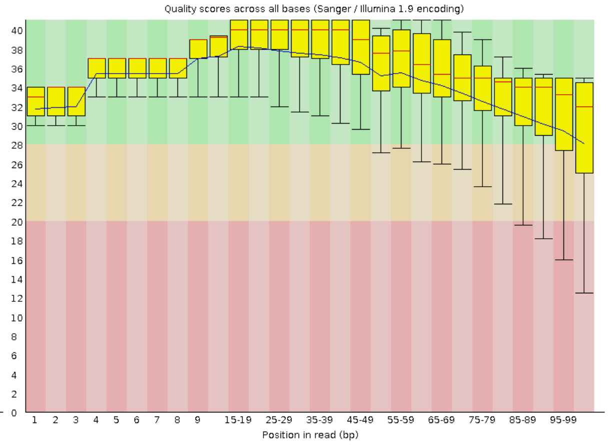

7.2 Per sequence quality scores

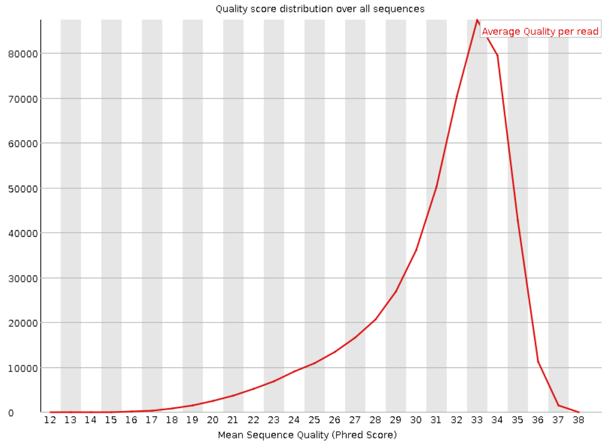

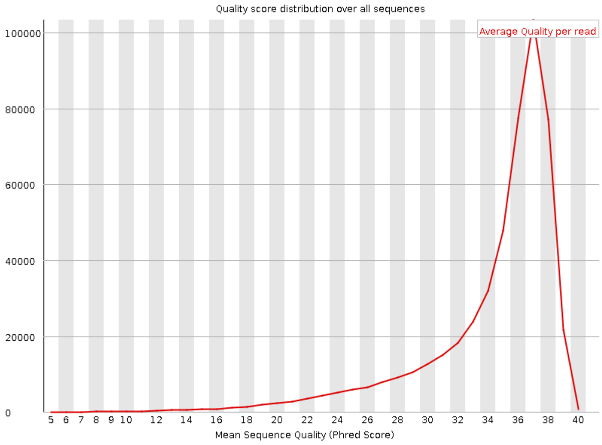

7.3 Per base sequence content

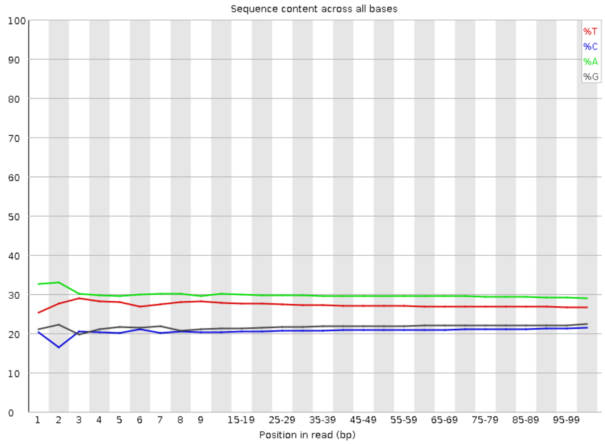

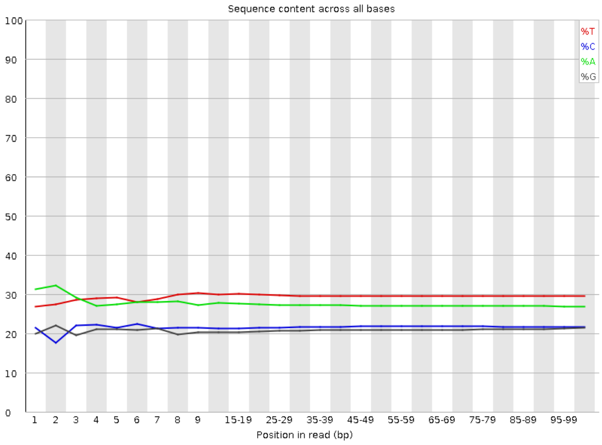

References

[1] Andrews, S.: Fastqc a quality control tool for high throughput sequence data. http://www.bioinformatics.babraham.ac.uk/projects/fastqc/ (2012)
