# Supplementary material for: UrQt: an efficient software for the Unsupervised Quality trimming of NGS data
Source: BMC Bioinformatics. 2015 Apr 29;16:137. doi: 10.1186/s12859-015-0546-8 (PMC4450468; doi:10.1186/s12859-015-0546-8)
Supplement: Additional file 3 — Mapping performances for the four tested programs. Mapping performances for different quality threshold with the four tested programs and the seven NGS sample (Table 1). Mapping results with Bowtie2 [17] and BWA [14]. [file 12859_2015_546_MOESM3_ESM.pdf]

Quality analysis of the seven NGS samples after trimming

December 22, 2014

Mapping performances for different quality thresholds with the four tested programs and the seven NGS sample (Table 1). Mapping results with Bowtie2 [1] and BWA [2]. The two programs were used with default parameters plus the `-very-sensitive` option for Bowtie2. Paired-end samples were mapped independently.

Table 1: **NGS data sets used for testing**

Accession number	Species	sample type	paired-end	read size (bp)	reference genome
SRR002073	*Homo sapiens*	RNA	no	33	hg19
SRR521463	*Homo sapiens*	RNA	yes	75	hg19
SRR420813	*Arabidopsis thaliana*	RNA	no	83	TAIR10
SRX150254	*Prunus persica*	DNA	yes	100	1.22
SRR452441	*Saccharomyces cerevisiae*	DNA	yes	100	EF4
SRR988074	*Drosophila melanogaster*	DNA	yes	101	5.41
SRR919326	*Drosophila melanogaster*	RNA	yes	101	5.41

The mapping were performed using the prebuild Bowtie2 and BWA indexes from the igenome project available at the following url:

- ftp://igenome:G3nom3s4u@ftp.illumina.com/Homo_sapiens/NCBI/build37.2/Homo_sapiens_NCBI_build37.2.tar.gz
- ftp://igenome:G3nom3s4u@ftp.illumina.com/Arabidopsis_thaliana/NCBI/TAIR10/Arabidopsis_thaliana_NCBI_TAIR10.tar.gz
- ftp://igenome:G3nom3s4u@ftp.illumina.com/Saccharomyces_cerevisiae/NCBI/build3.1/Saccharomyces_cerevisiae_NCBI_build3.1.tar.gz
- ftp://igenome:G3nom3s4u@ftp.illumina.com/Drosophila_melanogaster/NCBI/build5.41/Drosophila_melanogaster_NCBI_build5.41.tar.gz

Counts of reads mapping to unique or multiple positions correspond to count of the flags `XS` and `AS` for Bowtie2 and `XT:A:U` and `XT:A:R` for BWA.

Contents

1 Bowtie2 **4**
1.1 Reads mapped 4
1.2 Reads mapping to an unique position 5
1.3 Reads mapping to multiple positions 6

2 BWA **8**
2.1 Reads mapped 8
2.2 Reads mapping to an unique position 9
2.3 Reads mapping to multiple positions 10

1 Bowtie2

1.1 Reads mapped

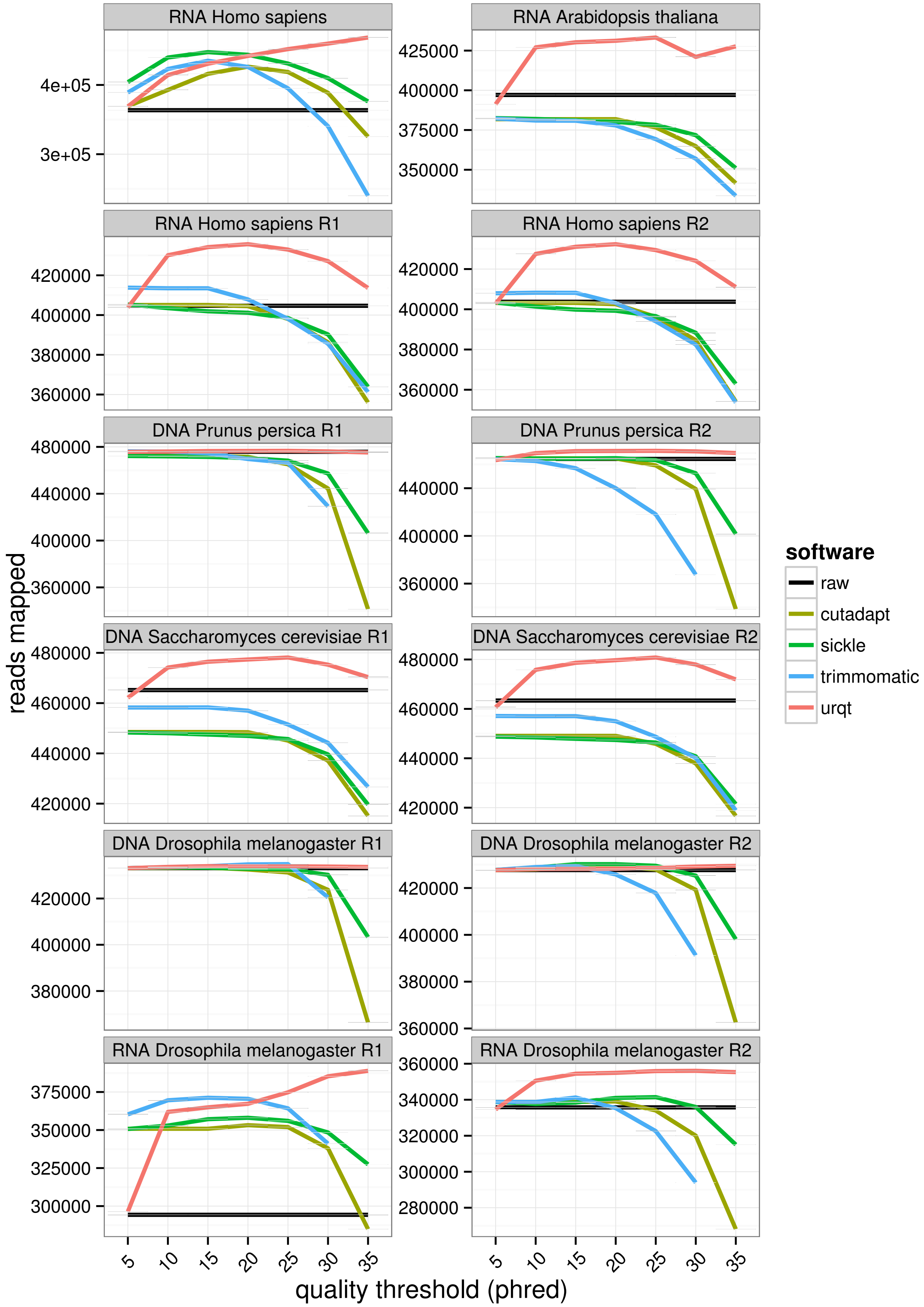

1.2 Reads mapping to an unique position

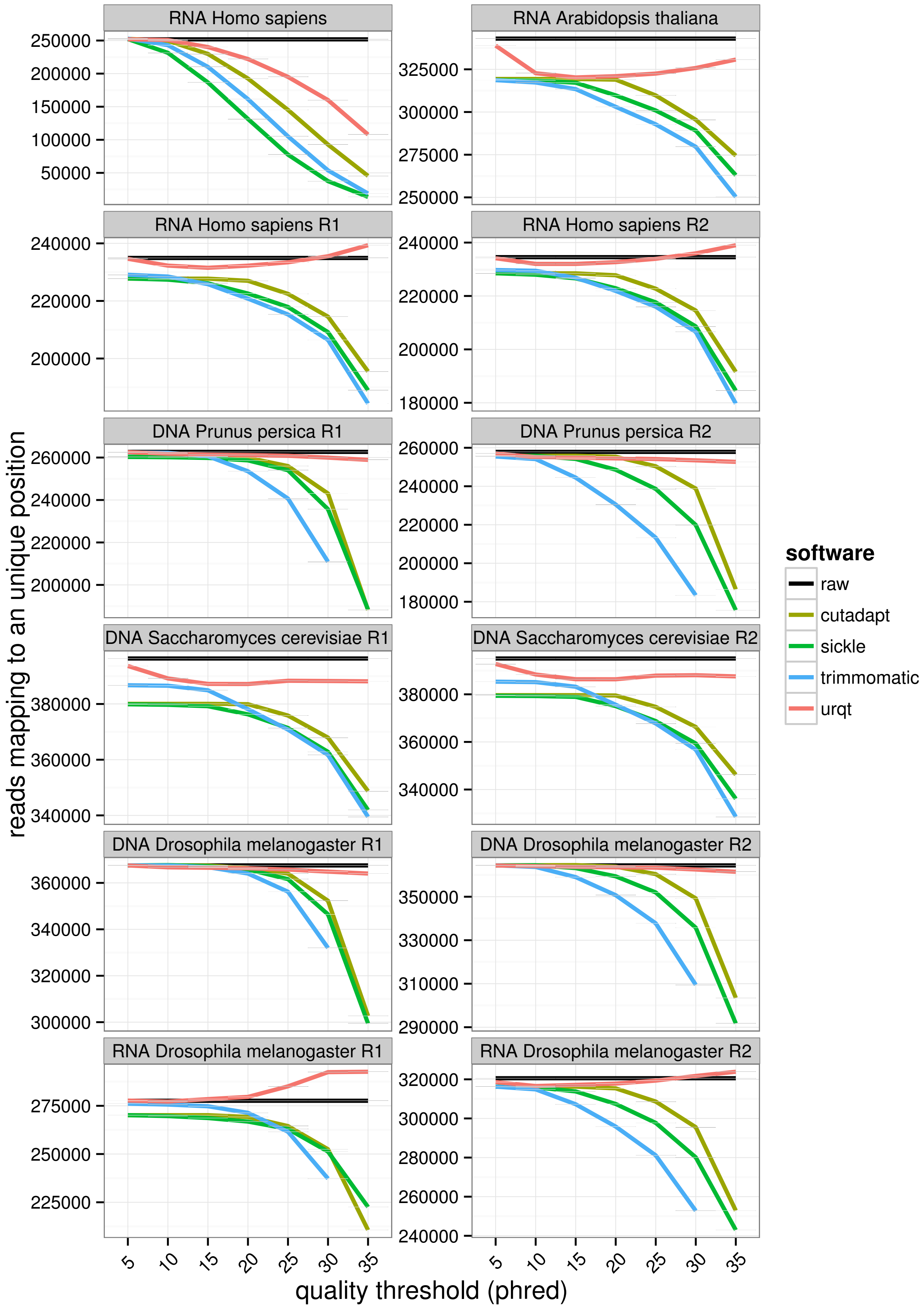

1.3 Reads mapping to multiple positions

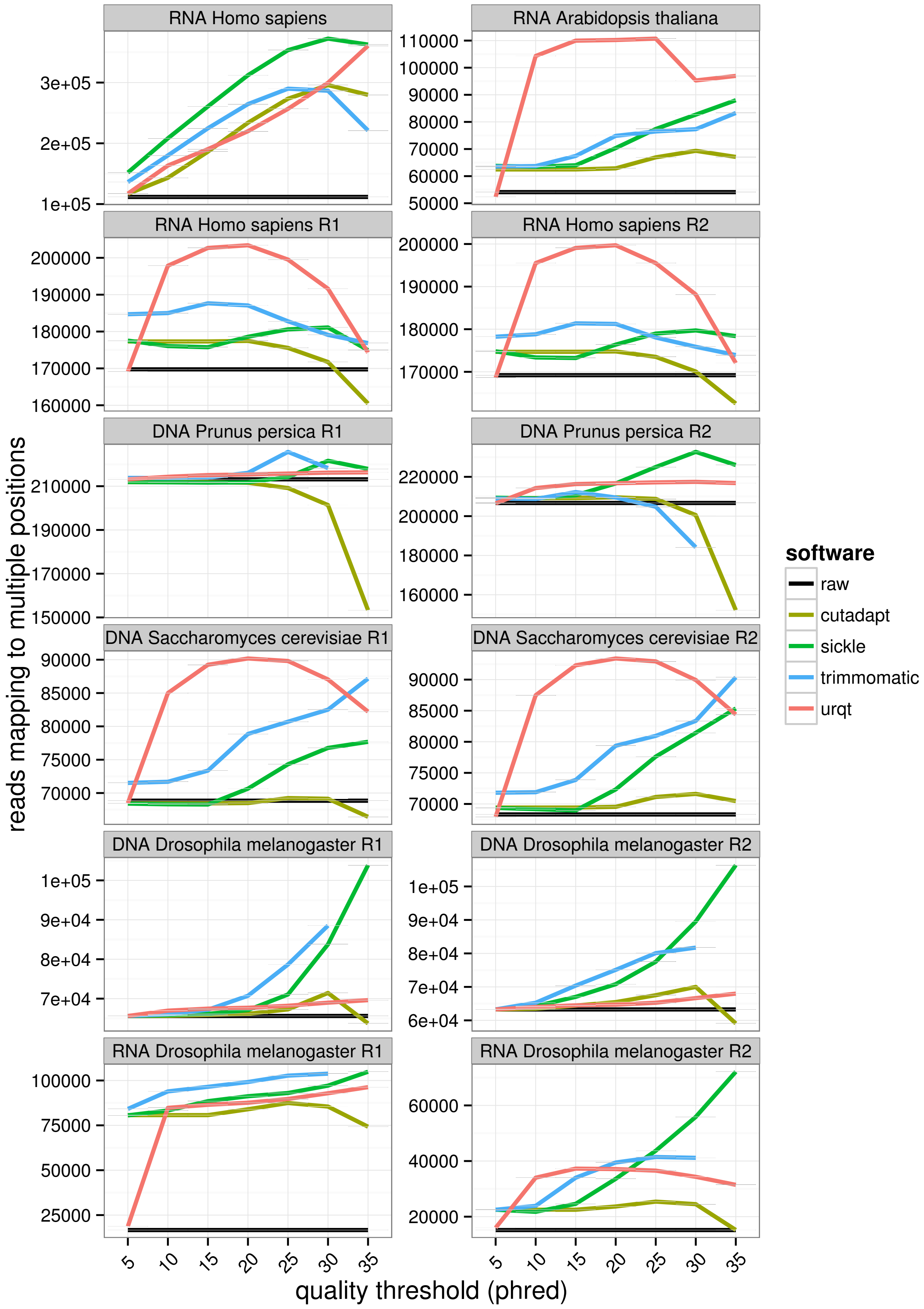

2 BWA

2.1 Reads mapped

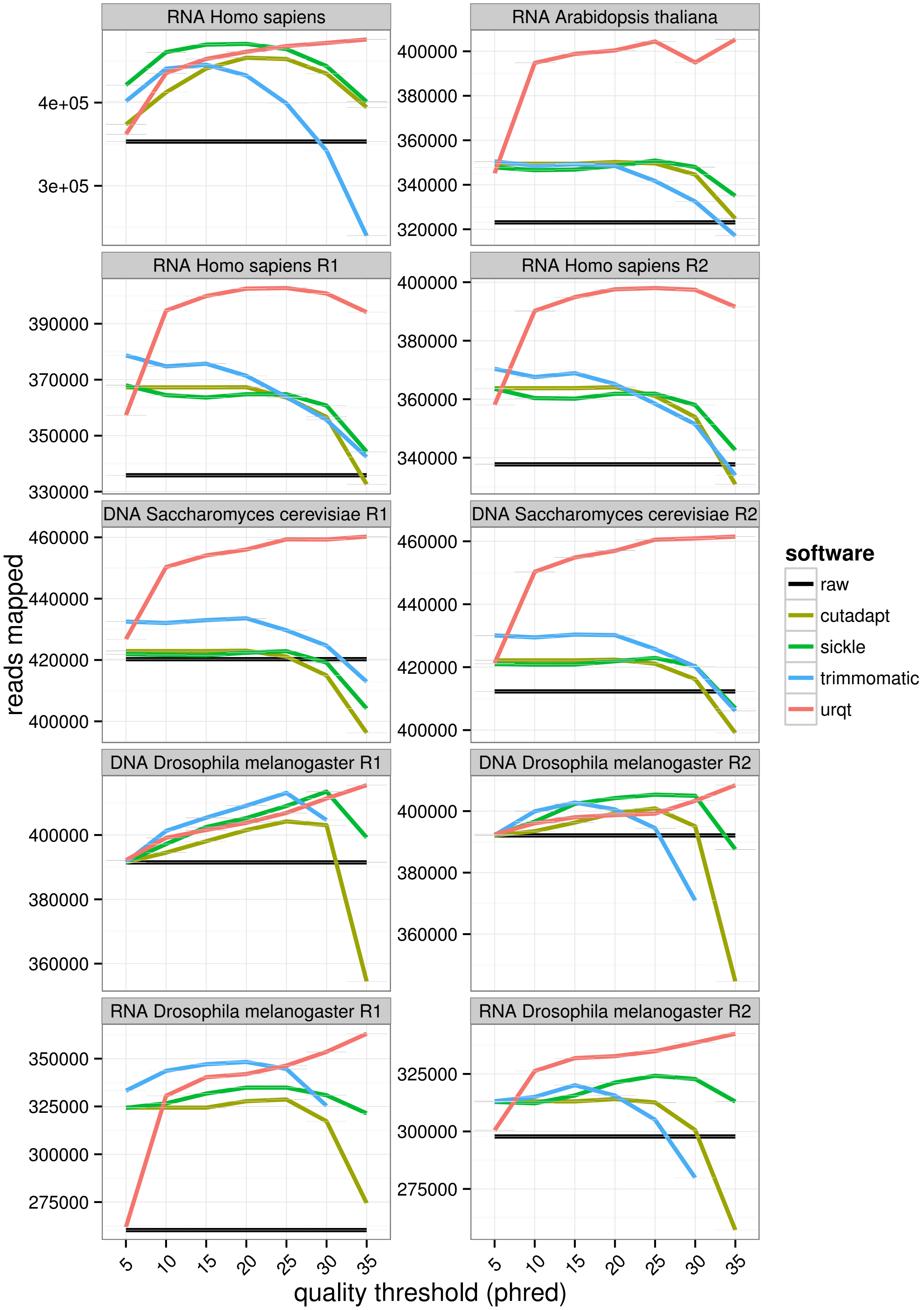

2.2 Reads mapping to an unique position

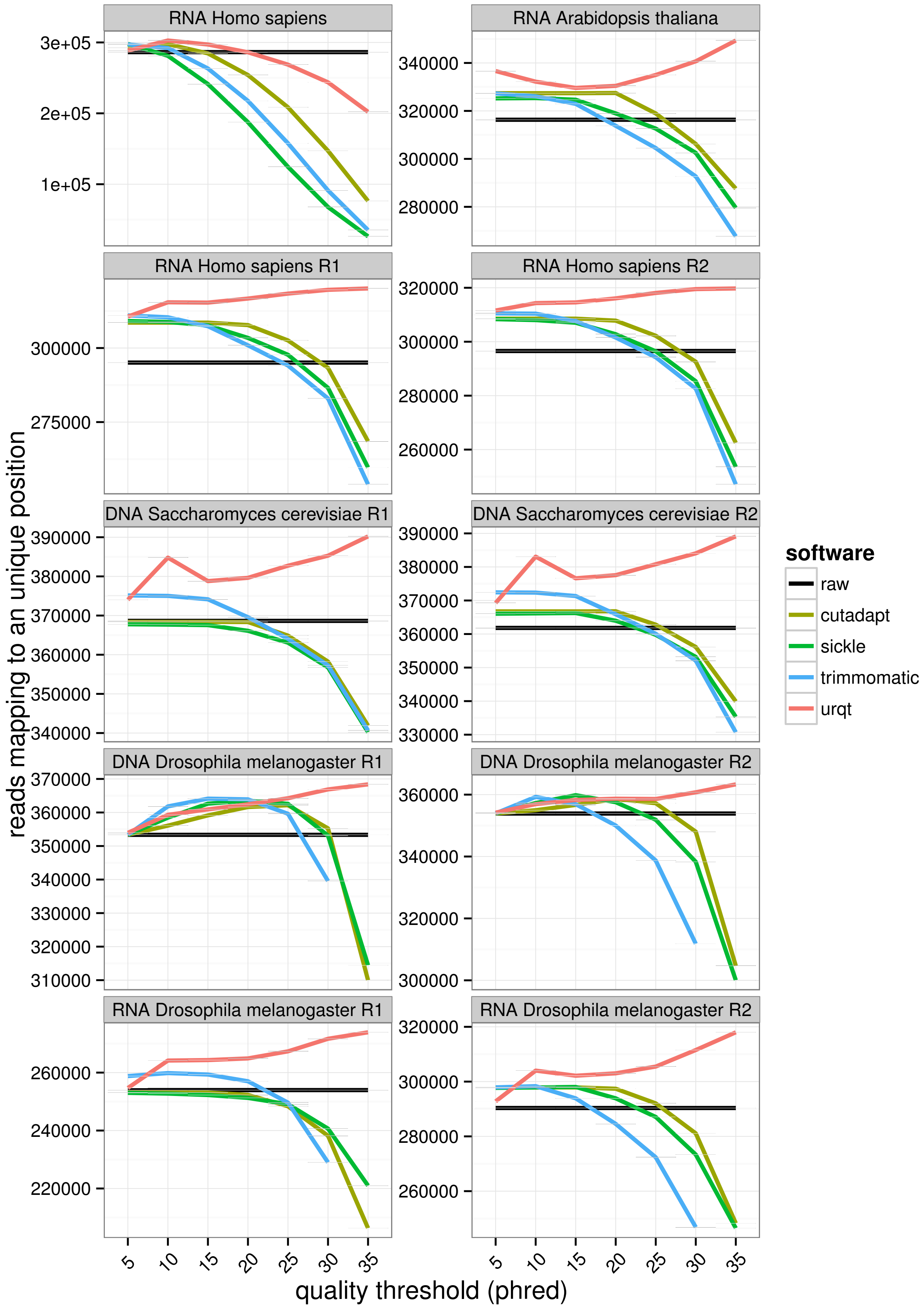

2.3 Reads mapping to multiple positions

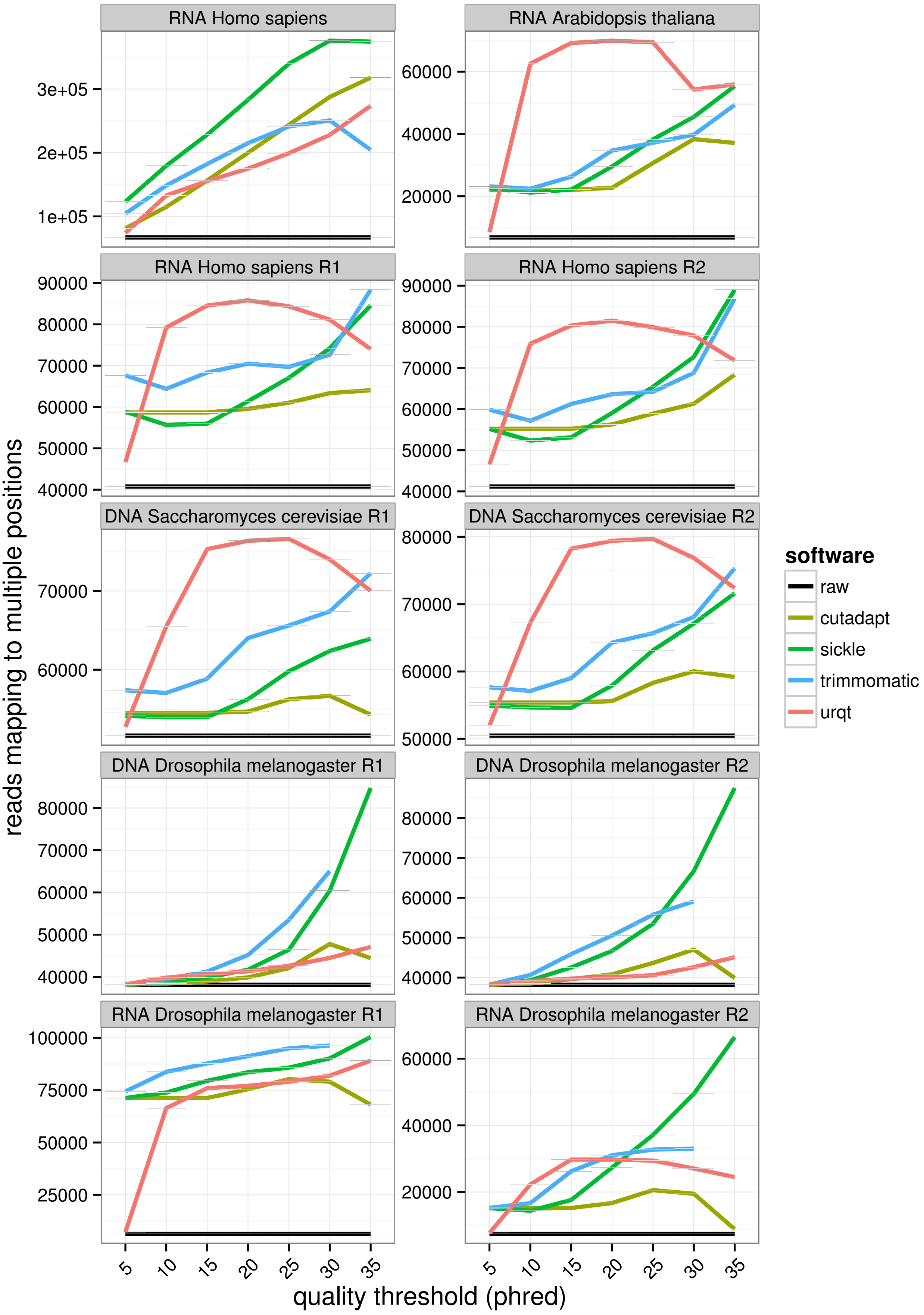

References

[1] Langmead, B., Salzberg, S.L.: Fast gapped-read alignment with Bowtie 2. Nat. Methods **9**(4), 357–9 (2012)

[2] Li, H., Durbin, R.: Fast and accurate short read alignment with burrows-wheeler transform. Bioinformatics **25**(14), 1754–1760 (2009). doi:10.1093/bioinformatics/btp324
